# Characterisation of hydration water in Nafion membrane†

**DOI:** 10.1039/d1ra00791b

**Published:** 2021-03-01

**Authors:** Stewart F. Parker, Shrey Shah

**Affiliations:** ISIS Facility, STFC Rutherford Appleton Laboratory Chilton, Didcot Oxon OX11 0QX UK stewart.parker@stfc.ac.uk

## Abstract

Nafion, a polytetrafluoroethylene polymer with perfluorinated-vinyl-polyether side chains ending in sulfonic acid groups, is widely used as the proton-exchange membrane in polymer electrolyte fuel cells, particularly low temperature hydrogen-oxygen fuel cells. The state of hydration of the sulfonic acid groups is crucial to its operation. By using a combination of inelastic neutron scattering (INS) and infrared spectroscopies, and by comparison to a series of trifluoromethanesulfonic acid hydrates of well-defined stoichiometry, we characterise how the hydration changes as a function of water content.

## Abstract 2

Hydration of fully dried Nafion membrane results in the formation of oxonium ions of increasing complexity, up to H_9_O_4_^+^. Beyond this, water behaves as the bulk liquid.

## Graphical abstract

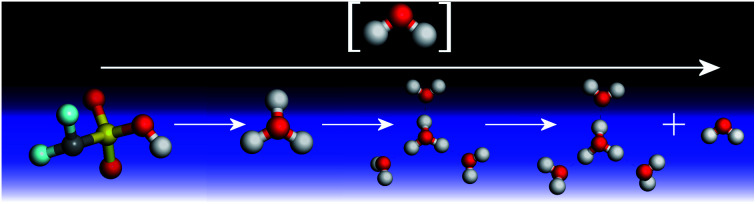

## Introduction

First manufactured in the 1970s by DuPont, Nafion^1^ is composed of a polytetrafluoroethylene (PTFE) backbone, with perfluorinated-vinyl-polyether side chains terminating in sulfonic acid groups. After synthesis, Nafion is cast into thin membranes by heating in aqueous alcohol at 250 °C in an autoclave.^2^ These membranes are characterised by both their thickness and equivalent weight (EW), which is the mass of dry Nafion per mole of sulfonic acid groups when the material is in acid form.^1^ For example, Nafion 112 has an equivalent weight of 1100 and a thickness of 0.002 inches.

Nafion is used as the proton-exchange membrane in polymer electrolyte fuel cells, particularly low temperature hydrogen-oxygen fuel cells, owing to its excellent ion conductivity, especially at low humidity, and chemical–mechanical stability, characteristic of perfluorinated polymers. These fuel cells are already being used in electric cars as they offer a compact, energy-dense alternative to conventional lithium-ion battery cells. It is not, however, the perfect material for these applications, as it is expensive and has high gas permeability, as well as low environmental compatibility.^3^ It is important to understand the reason for Nafion's high proton conductivity at low humidity, so that different proton exchange membranes can be developed which do not have the drawbacks of Nafion but still function well at low humidity.

Because of its commercial importance, Nafion has been the subject of extensive research. Knowledge of the state of water in the membrane is of crucial importance to understanding its role and properties. Vibrational spectroscopy has played a major role in these studies.^3–9^ Typically, attenuated total internal reflection infrared (ATR-IR) spectroscopy is preferred over Raman spectroscopy, as the Nafion membranes often fluoresce, even with near infrared excitation. Moreover, Raman spectroscopy is much less sensitive to water and related species than IR. However, it is sometimes beneficial to use Raman and ATR-IR in conjunction, as even the thinnest Nafion membranes are totally absorbing with respect to infrared light, however, the focal point of the Raman laser can be adjusted to look at any specific area within the material. This means that while ATR-IR spectroscopy is limited to looking at water on the surface of the membrane, Raman spectroscopy can be used to investigate water within the membrane. Both techniques are well established, so data can be obtained relatively quickly and easily.

In this paper, inelastic neutron scattering (INS) spectroscopy has been used in combination with ATR-IR to determine the structure of water within the wetted Nafion membranes. INS spectroscopy is a form of vibrational spectroscopy conceptually similar to Raman spectroscopy, however instead of a photon being inelastically scattered, neutrons are used. The intensity,^10^*S*, of an INS band is given by:1
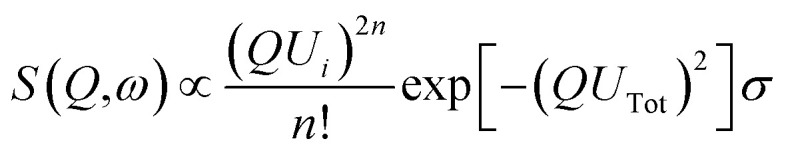
where *i* is the *i*th mode at frequency *ω*, *n* = 1 for a fundamental, 2 for a first overtone or binary combination, 3 for a second overtone or ternary combination and so on. *Q* is the momentum transfer defined as: *Q* = *k*_*i*_ − *k*_f_ where *k* = 2π/*λ*, (*k* is the wavevector (Å^−1^), *λ* (Å) is the wavelength of the neutron). *U*_*i*_ is the root mean square displacement of the atoms in the mode. The exponential term is a Debye–Waller factor, *U*_Tot_ is the total root mean square displacement of all the atoms in all the modes, internal and external. To minimise the Debye–Waller factor, INS spectra are usually measured below 20 K. *σ* is the scattering cross section of the scattering atom.

^1^H has a particularly large incoherent scattering cross-section of 80.3 barn, whereas most other elements sit at <5 barn.^11^^1^H is also the lightest isotope of the lightest element, and consequently has the largest amplitude of vibration. Therefore, for materials containing hydrogen, incoherent scattering due to hydrogen dominates the spectrum. For this reason, INS is the ideal technique to study water within Nafion, as the perfluorinated components are barely visible in the resulting spectra.

One approach in determining the state of water in Nafion is to investigate the structure of model compounds. A simple model of the sulfonic acid functionality is trifluoromethanesulfonic acid (CF_3_SO_3_H, or TFSA). This forms a series of structurally characterised hydrates: TFSA·*n*H_2_O (*n* = 0,^12^ 0.5,^13^ 1,^14,15^ 2,^13^ 4,^16^ 5 ^27^ ), (the phase diagram^13^ is reproduced in Fig. S1†), which contain progressively more complex forms of protonated water (oxonium ions) ranging from just the sulfonic acid functionality (*n* = 0), to hydroxonium ions (*n* = 1), H_5_O_2_^+^ ions (*n* = 2), to H_9_O_4_^+^ ions (*n* = 4 and 5). The *n* = 0.5 material contains a 1 : 1 mixture of sulfonic acid and hydroxonium ions. The fifth water molecule in the *n* = 5 compound links the H_9_O_4_^+^ ions.

In this paper we are leveraging the selectivity of neutron scattering to hydrogen vibrations to characterise the state of water in Nafion as a function of hydration. To this end, we use TFSA hydrates as model compounds of known stoichiometry to determine the state of water as the hydration level varies.

## Experimental

Nafion™ N-117 membrane (0.18 mm thickness, >0.90 meq g^−1^ exchange capacity, lot no. W27E078) was purchased from Alfa Aesar. Samples with different water contents were prepared as shown in Table 1. The water content was determined gravimetrically, assuming that the 180 °C dried sample was water-free (this was confirmed by the spectra).

**Table 1:** Preparation of Nafion samples with different water contents

Preparation method	Water content/wt%	Moles H_2_O per SO_3_
Dried in vacuum oven at 180 °C for two days	0	0
Dried in glove box at room temperature for seven days	1.51	1.0
Dried over P_2_O_5_ for 6 days	2.46	1.6
Dried over MgCl_2_ for 6 days	6.10	4.0
Used as received	7.33	4.8
Soaked in distilled water for three days	19.39	12.7

Trifluoromethanesulfonic acid (≥99%) was purchased from Sigma-Aldrich and used as received. The TFSA hydrates were made by carefully mixing stoichiometric amounts of TFSA and distilled water under a flow of inert gas.

INS spectra were recorded using the TOSCA^17^ and MAPS^11,18^ spectrometers at the ISIS Pulsed Neutron and Muon Facility (Chilton, Oxfordshire, UK).^19^ The two instruments are complementary: on TOSCA, the resolution is 1.25% of the energy transfer across the entire energy range, thus provides high resolution spectra in the 0–2000 cm^−1^ range, however, instrumental factors^10,11^ mean that there is little information at >2000 cm^−1^. MAPS provides access to the O–H stretch region with modest resolution. The INS spectra shown have been normalised to 1 g of Nafion or 1 g of TFSA. The TOSCA spectra are available from the INS database at: http://wwwisis2.isis.rl.ac.uk/INSdatabase/. Infrared spectra (4 cm^−1^ resolution, 64 scans) were recorded between 105 K and 298 K with a Bruker Vertex 70 Fourier transform infrared spectrometer using a Specac single reflection variable temperature attenuated total internal reflection accessory. FT-Raman spectra were recorded with a Bruker MultiRam spectrometer using 1064 nm excitation, 4 cm^−1^ resolution, 500 mW laser power and 64 scans at room temperature.

## Results and discussion

**Fig. 1:**
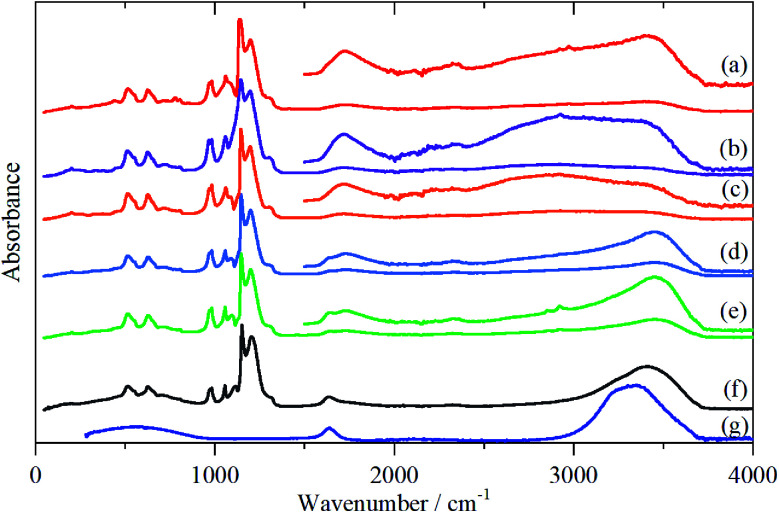
Infrared spectra of Nafion at various levels (wt%) of hydration: (a) 0, (b) 1.51, (c) 2.46, (d) 6.10, (e) 7.33, (f) 19.39 and (g) liquid water. The insets in (a)–(e) show the 1500–4000 cm^−1^ region ordinate expanded ×5 (a)–(c) and ×3 (d), (e) relative to the 0–4000 cm^−1^ region.

**Fig. 2:**
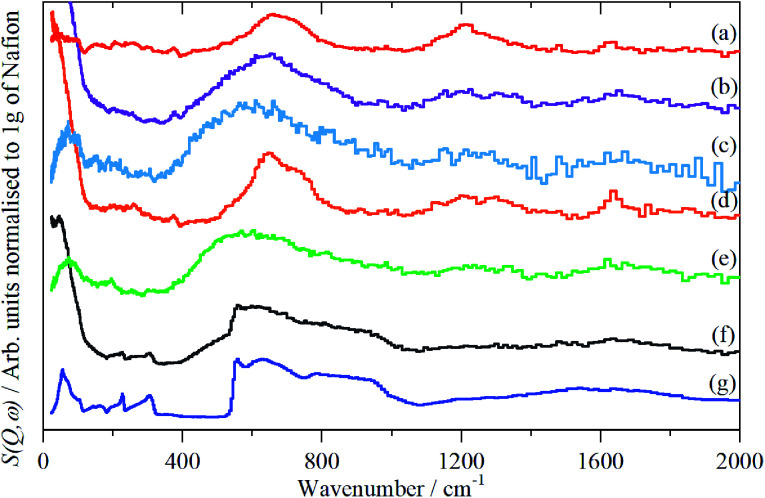
INS (TOSCA) spectra of Nafion at various levels (wt%) of hydration: (a) 0, (b) 1.51, (c) 2.46, (d) 6.10, (e) 7.33, (f) 19.39 (g) ice I_h_.

**Fig. 3:**
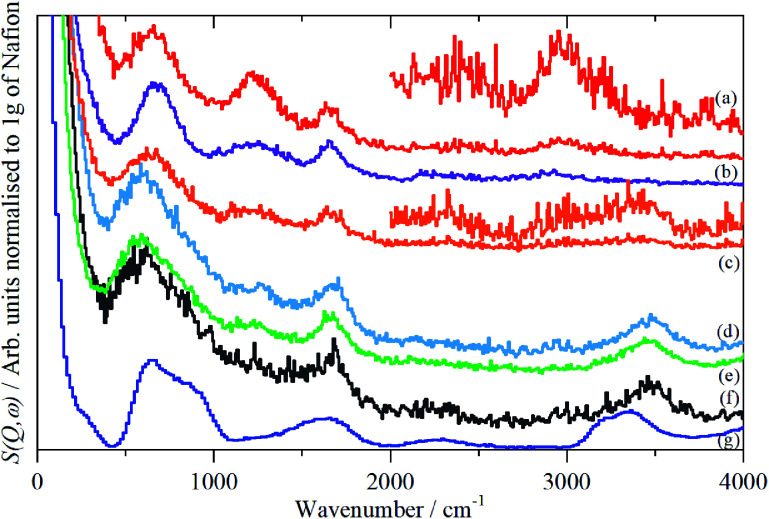
INS (MAPS) spectra of Nafion at various levels (wt%) of hydration: (a) 0, (b) 1.51, (c) 2.46, (d) 6.10, (e) 7.33, (f) 19.39 (g) ice I_h_. The insets in (a) and (c) show the 2000–4000 cm^−1^ region ordinate expanded ×5 (a) and ×4 (c), (e) relative to the 0–4000 cm^−1^ plot.

The infrared spectra of Nafion at various degrees of hydration are shown in Fig. 1. These are in good agreement with previous work.^5,8^ As expected, the region below 1500 cm^−1^ is dominated by the modes of the perfluoro skeleton and only the H–O–H bending and O–H stretch modes of water are clearly seen; the librational and translational modes are unobserved. In contrast, in the INS spectra, Fig. 2 and 3, all of the water and oxonium-related modes are apparent, the perfluoro skeletal modes are absent. This arises because the INS spectra are dominated by modes that involve significant displacement of hydrogen atoms. Note that only motion is required; thus for water the librational modes at 500–1000 cm^−1^ exhibit high intensity, Fig. 2g, even though there is (almost) no change in the H–O–H angle or O–H distances as the mode is executed.

There is a marked difference in the appearance of the O–H stretch modes between the infrared and INS spectra, Fig. S2–S7.† A degree of caution is required as the infrared spectra are measured by ATR-IR, so are representative of the surface material, whereas the INS spectra are an average of the entire (surface + bulk) film, where the bulk dominates. The infrared spectra are strongly affected by electrical anharmonicity, which results in the O–H stretch bands being extremely broad, this is especially noticeable for the low hydration spectra (Fig. 1a–c) where the bands are ∼800 cm^−1^ wide, whereas the widths in the corresponding INS spectra (Fig. 3a–c) are half this.

Comparison of the infrared and INS spectra allows some trends to be picked out. As the hydration level increases, the O–H stretch modes move to higher energy. In the most hydrated samples, these occur above the modes of water or ice I_h_, showing that the hydrogen bonding is weaker than in water. The TOSCA spectra (Fig. 2) show that at all, except for the highest, hydration levels, the water (and/or oxonium ions) are present as disordered materials, as shown by the absence of structure in the translational (0–300 cm^−1^) and librational (500–1000 cm^−1^) modes. In contrast, at the highest hydration level (Fig. 2f) water (ice) is present in both ordered *i.e.* ice I_h_ and disordered forms, as may be seen by comparison of Fig. 2f with 2e and g: 2f is clearly a superposition of the two.

**Fig. 4:**
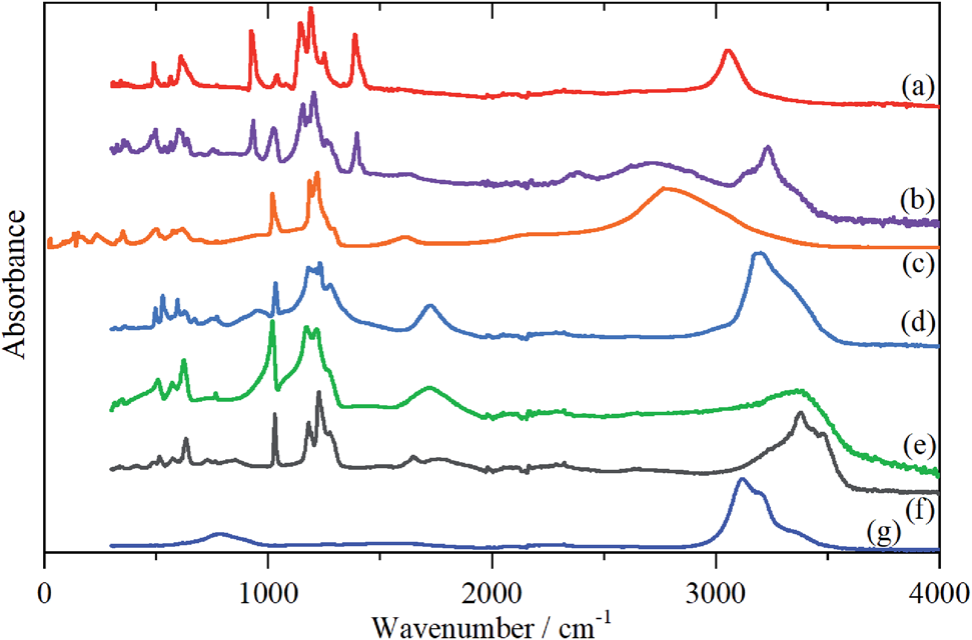
Solid state infrared spectra of TFSA·*n*H_2_O: (a) *n* = 0 at 173 K, (b) *n* = 0.5 at 158 K, (c) *n* = 1 at 299 K, (d) *n* = 2 at 158 K, (e) *n* = 4 at 175 K, (f) *n* = 5 at 200 K, (g) ice I_h_ at 200 K.

**Fig. 5:**
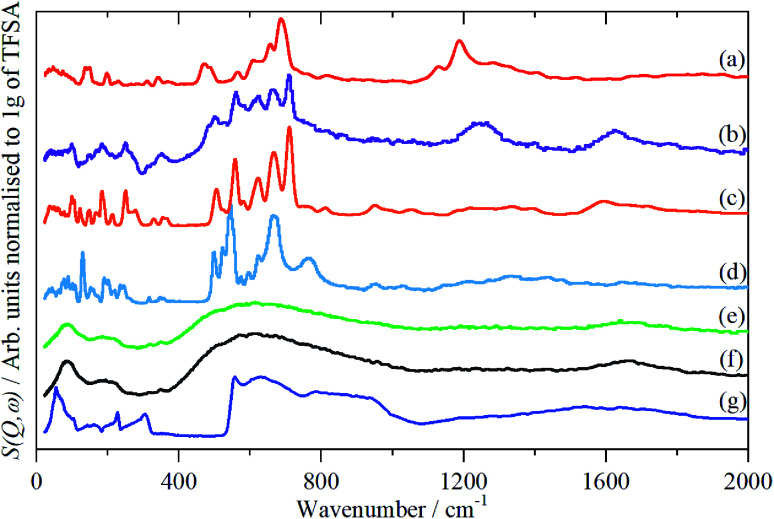
INS (TOSCA) spectra of TFSA·*n*H_2_O at <20 K: (a) *n* = 0, (b) *n* = 0.5, (c) *n* = 1, (d) *n* = 2, (e) *n* = 4, (f) *n* = 5, (g) ice I_h_. Relative to (d), (e) and (f); (a) is ×6, (b) is ×3 and (c) ×2 ordinate expanded.

**Fig. 6:**
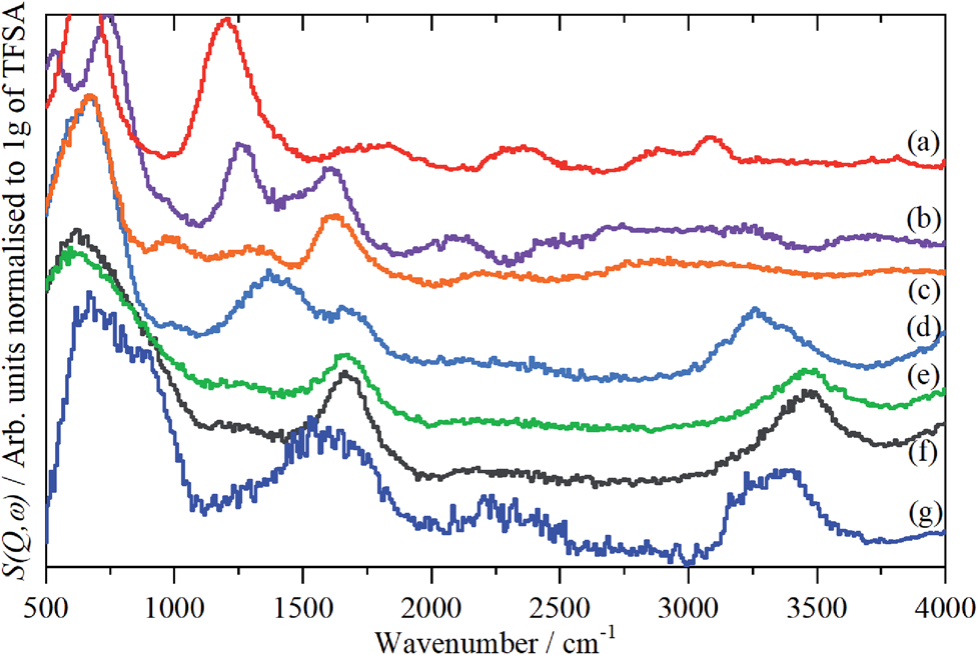
INS (MAPS) spectra of TFSA.*n*H_2_O at <20 K: (a) *n* = 0, (b) *n* = 0.5, (c) *n* = 1, (d) *n* = 2, (e) *n* = 4, (f) *n* = 5, (g) ice I_h_. Relative to (d)–(f); (a)–(c) ×2 ordinate expanded.

In order to aid the assignment of the Nafion spectra, we have also recorded the spectra of a series of model compounds, TFSA·*n*H_2_O, that have crystallographically characterised forms of oxonium, Fig. 4–6. These have melting points at: 233, 258, 307.5, 267, 225 and 227 K for *n* = 0, 0.5, 1, 2, 4 and 5 respectively (see Fig. S1†^13^). Thus all of them, except for the monohydrate, are liquids at room temperature. As may be expected, there are significant changes in the infrared spectra between the liquid and solid state, particularly for the O–H stretch modes (see Fig. S8–S19†). As the INS spectra are recorded below 20 K, the solid state infrared spectra are shown in Fig. 4.

As with Nafion, the infrared spectra below 1500 cm^−1^ are dominated by skeletal modes, in this case of the [CF_3_SO_3_]^−^ ion, Fig. 4, as are the Raman spectra (where they are available, Fig. S11, S12, S15, S17, S19†). In the infrared spectra, there is little change between the liquid and solid states for the [CF_3_SO_3_]^−^ ion modes. Comparison of Fig. 4a–c and 5a–c show that, as might be expected, the spectrum of the *n* = 0.5 compound is a simple summation of the spectra of the parent acid and that of the monohydrate.

The availability of both the infrared and the INS spectra enables the key features of the various species to be assigned by inspection. The results are given in Table 2. From this, and the spectra, several trends are apparent. As the oxonium ion becomes larger, the O–H stretch shifts to higher energy. This is a reflection of the weaker hydrogen bonding present between water and the central H_3_O^+^ ion than that between sulfonate and the ion. Thus, in the *n* = 1 compound, there are three H-bonds to sulfonate; in the *n* = 2 there are two and one to water; in *n* = 4 and 5 there are none to sulfonate and three to water. The asymmetric bending mode also shifts to higher energy as *n* increases. In the infrared spectrum of the *n* = 2 compound (Fig. 5d), a shoulder is apparent on the low energy side of the asymmetric bending mode, this is more apparent in the *n* = 4 and 5 compounds. This is assigned to the bending mode of the H-bonded water molecules. All of the spectra of the *n* = 4 and 5 compounds are strikingly similar, suggesting that the additional water molecule only causes a minor perturbation to the H_9_O_4_^+^ ion found in the *n* = 4 material.

**Table 2:** O–H related modes (cm^−1^) in ice and TFSA·*n*H_2_O

Description	Species				
	Ice	*n* = 0	*n* = 1 (H_3_O^+^)	*n* = 2 (H_5_O_3_^+^)	*n* = 4 (H_9_O_4_^+^)
O–H stretch	3215, 3380	3090	2850	3260, 3380	3460
O–H bend	1580	685 (oop), 1185 (ip)^a^	950 (sym), 1616 (asym)^b^	1640 (sh),^c^ 1730	1644, 1746
Librations	535–1000		480–830	470–810	370–1040
Translations	20–325		20–300	20–270	20–260

a oop = out-of-plane S–O–H bend, ip = in-plane S–O–H bend.

b asym = asymmetric bend, sym = symmetric bend.

c sh = shoulder.

The translational (0–300 cm^−1^) and librational (450–1000 cm^−1^) modes of water in ice and the *n* = 0.5, 1 and 2 compounds give sharp bands, as expected for ordered structures. In contrast, the bands of the *n* = 4 and 5 compounds are broad with little structure and are much more characteristic of those seen for disordered water on surfaces.^20–22^

From the ratio of water to sulfonic acid in Table 1 and by knowing how the protons are organised in the TFSA·*n*H_2_O compounds, we can deduce how the water is present in the Nafion samples. Thus Nafion dried at 180 °C would be expected to only contain sulfonic acid groups; the samples dried in the glove box and over P_2_O_5_ would contain only H_3_O^+^ ions; the as-received and dried over MgCl_2_ samples would contain largely H_9_O_4_^+^ ions and the sample soaked in water would be a mixture of H_9_O_4_^+^ ions and water.

**Fig. 7:**
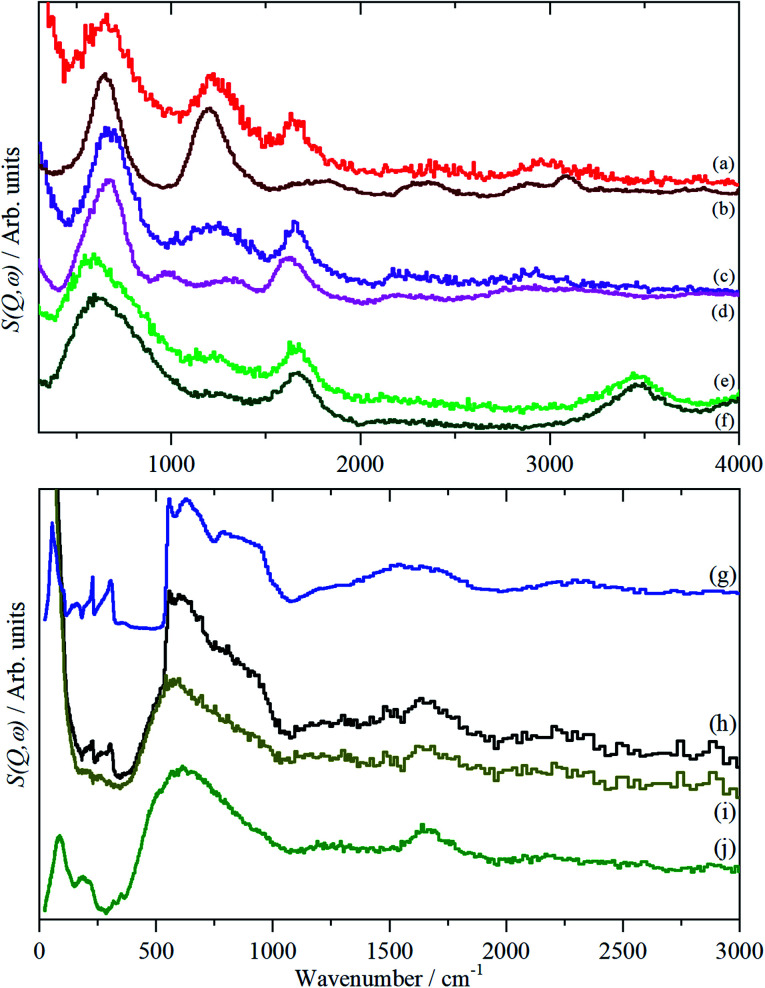
Comparison of INS spectra of Nafion at different hydration levels and TFSA·*n*H_2_O: (a) Nafion dried at 180 °C, (b) *n* = 0, (c) Nafion dried in the glovebox, (d) *n* = 1, (e) Nafion as received, (f) *n* = 4, (g) ice I_h_, (h) Nafion soaked in water, (i) water subtracted difference spectrum *i.e.* (h)–(g) and (j) *n* = 4. (a) to (f) are recorded with MAPS and (g) to (j) with TOSCA. (h) and (i) are plotted on the same scale.

A comparison of the spectra of Nafion with the appropriate model compound supports these assignments, Fig. 7. The infrared and INS spectra of Nafion support these assignments. In the infrared spectra, Fig. 1a–c, the broad bands at 2500–3000 cm^−1^ are characteristic of the electrical anharmonicity caused by the strong hydrogen-bonding present with the H_3_O^+^ ion, (we believe that its presence in the fully dried material, Fig. 1a, is the result of re-adsorption of water by the sulfonic acid groups at the surface, as the INS spectrum, Fig. 3a, which is a bulk measurement, shows only sulfonic acid). As the hydration levels increase, the O–H stretch bands move to higher energy as seen for the TFSA·*n*H_2_O compounds.

Compilations of each type of spectra recorded are presented in the ESI†: Nafion Fig. S2–S7† and TFSA and its hydrates in Fig. S8–S17.†

## Conclusions

In this work we have characterised the state of hydration of a Nafion membrane as a function of water content by comparison to series of model compounds of defined structure.

It is surprisingly difficult to dehydrate Nafion: an extended period at high temperature is required to remove all the water and just leave the sulfonic acid groups. Hydroxonium, H_3_O^+^, groups are present at low water contents. For the as received membrane, water is present as a complex oxonium ion, H_9_O_4_^+^. Addition of more water molecules by immersion in water does not change this ion, rather the additional water is present as unperturbed water molecules. This is shown by the formation of ice I_h_. Subtraction of the ice contribution to the spectrum, Fig. 7i, shows that approximately 50% of the water is present in this form and this is presumably also the case for the working material.

The results presented here support the current models of proton transport in Nafion (reviewed in ref. 23). These show that at low hydration levels, the conductivity is largely by diffusion of H_3_O^+^ ions, above 5 wt% H_2_O molecular dynamics^24^ studies show that the H_9_O_4_^+^ ion becomes increasingly important and that the conduction mechanism is dominated by Grotthuss hopping between ions. Our results show that below 5 wt%, only H_3_O^+^ ions are present, at higher water concentrations the H_9_O_4_^+^ ion is present and at saturation, unbound water is also present, that facilitates the Grotthuss proton transport mechanism.

## Conflicts of interest

There are no conflicts to declare.

## Supplementary Material

RA-011-D1RA00791B-s001

## References

[cit1] Mauritz K. A., Moore R. B. (2004). Chem. Rev..

[cit2] Heitner-Wirquin C. (1996). J. Membr. Sci..

[cit3] Kunimatsu R. S. K., Miyatake K., Tsuneda T. (2016). Macromolecules.

[cit4] Ostrovskii D., Brodin A., Torell L. (1996). Solid State Ionics.

[cit5] Gruger A., Régis A., Schmatko T., Colomban P. (2001). Vib. Spectrosc..

[cit6] Waren D., McQuillan A. (2008). J. Phys. Chem. B.

[cit7] Hofmann D., Kuleshova L., D'Aguanno B., Noto V. D., Negro E., Conti F., Vittadello M. (2009). J. Phys. Chem. B.

[cit8] Morita S., Kitagawa K. (2010). J. Mol. Struct..

[cit9] Aquino A., Tunega D. (2017). J. Phys. Chem. C.

[cit10] Mitchell P. C. H. , Parker S. F., Ramirez-Cuesta A. J. and Tomkinson J., Vibrational spectroscopy with neutrons, with applications in chemistry, biology, materials science and catalysis, World Scientific, Singapore, 2005

[cit11] Parker S. F., Lennon D., Albers P. (2011). Appl. Spectrosc..

[cit12] Bartmann K., Moot D. (1990). Acta Crystallogr., Sect. C: Cryst. Struct. Commun..

[cit13] Delaplane R., Lundgren J., Olovsson I. (1975). Acta Crystallogr., Sect. B: Struct. Sci., Cryst. Eng. Mater..

[cit14] Spencer J., Lundgren J. (1973). Acta Crystallogr., Sect. B: Struct. Crystallogr. Cryst. Chem..

[cit15] Lundgren J., Tellgren R., Olovsson I. (1978). Acta Crystallogr., Sect. B: Struct. Crystallogr. Cryst. Chem..

[cit16] Lundgren J. (1978). Acta Crystallogr., Sect. B: Struct. Crystallogr. Cryst. Chem..

[cit17] Pinna R., Rudić S., Parker S. F., Armstrong J., Zanetti M., Škoro G., Waller S. P., Zacek D., Smith C. A., Capstick M. J., McPhail D. J., Pooley D. E., Howells G. D., Gorini G., Fernandez-Alonso F. (2018). Nucl. Instrum. Methods Phys. Res., Sect. A.

[cit18] Ewings R. A., Stewart J. R., Perring T. G., Bewley R. I., Le M. D., Raspino D., Pooley D. E., Škoro G., Waller S. P., Zacek D., Smith C. A., Riehl-Shaw R. C. (2019). Rev. Sci. Instrum..

[cit19] https://www.isis.stfc.ac.uk/Pages/home.aspx

[cit20] Wang Y., Dong S. (2003). Phys. Rev. B: Condens. Matter Mater. Phys..

[cit21] Wang H.-W., DelloStritto M. J., Kumar N., Kolesnikov A. I., Kent P. R. C., Kubicki J. D., Wesolowski D. J., Sofo J. O. (2014). J. Phys. Chem. C.

[cit22] Parker S. F., Robertson S. J., Imberti S. (2019). Mol. Phys..

[cit23] Kusoglu A., Weber A. Z. (2017). Chem. Rev..

[cit24] Cui S., Liu J., Selvan M. E., Keffer D. J., Edwards B. J., Steele W. V. (2007). J. Phys. Chem. B.

[cit25] Parker S. F. , STFC, ISIS Neutron Muon Source, 2018, 10.5286/ISIS.E.RB1910022

[cit26] Parker S. F. , STFC, ISIS Neutron Muon Source, 2019, 10.5286/ISIS.E.RB1920052

[cit27] Lundgren J. (1978). Acta Crystallogr., Sect. B: Struct. Crystallogr. Cryst. Chem..

