# Supplementary material for: Characterisation of hydration water in Nafion membrane
Source: RSC Adv. 2021 Mar 1;11(16):9381–5. doi: 10.1039/d1ra00791b (PMC8695510; doi:10.1039/d1ra00791b)
Supplement: RA-011-D1RA00791B-s001 [file RA-011-D1RA00791B-s001.pdf]

Electronic Supplementary Material (ESI) for RSC Advances.
This journal is © The Royal Society of Chemistry 2021

Electronic Supplementary Information
for:

Characterisation of hydration water in Nafion membrane

Stewart F. Parker* and Shrey Shah, ISIS Facility, STFC Rutherford Appleton Laboratory, Chilton, Didcot, Oxon, OX11 0QX, UK.

Contents

S1 - Phase diagram of TFSA.*n*H_2O **2**

S2 - Infrared and INS spectra of Nafion dried 180 ˚C **3**

S3 - Infrared and INS spectra of Nafion dried in a glove box **3**

S4 - Infrared and INS spectra of Nafion dried over P_2O_5 .... **4**

S5 - Infrared and INS spectra of Nafion dried over $MgCl_2$ **4**

S6 - Infrared and INS spectra of Nafion as received.......... **5**

S7 - Infrared and INS spectra of Nafion soaked in water.......... **5**

S8 – Solid state infrared and INS spectra of TFSA **6**

S9 - Infrared spectra of liquid and solid TFSA.......... **6**

S10 - Solid state infrared and INS spectra of TFSA.0.5H_2O **7**

S11 - Spectra of liquid and solid TFSA.0.5H_2O **7**

S12 - Solid state infrared, FT-Raman and INS spectra of TFSA.H_2O **8**

S13 - Infrared spectra of liquid and solid TFSA.H_2O. **8**

S14 - Solid state infrared and INS spectra of TFSA.2H_2O **9**

S15 - Spectra of liquid and solid TFSA.2H_2O **9**

S16 - Solid state infrared and INS spectra of TFSA.4H_2O **10**

S17 - Spectra of liquid and solid TFSA.4H_2O **10**

S18 - Solid state infrared and INS spectra of TFSA.5H_2O **11**

S19 - Spectra of liquid and solid TFSA.5H_2O **11**

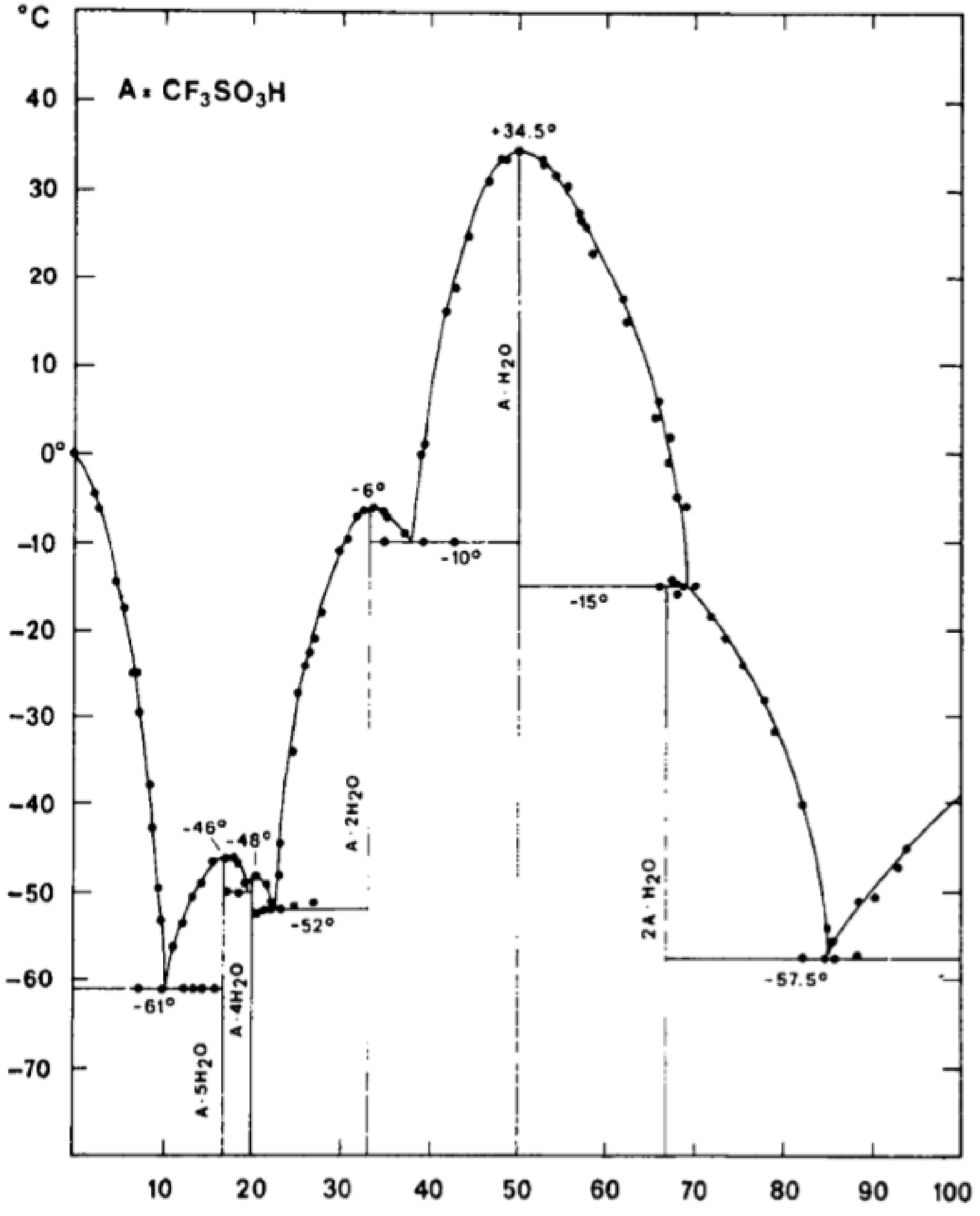

Fig. S1 Phase diagram of TFSA.$n$$H_2O$. Reproduced from: R. Delaplane, J. Lundgren and I. Olovsson, *Acta Cryst*. B31 (1975) 2208–2213 (doi: 10.1107/S056774087500725X) with permission of the International Union of Crystallography.

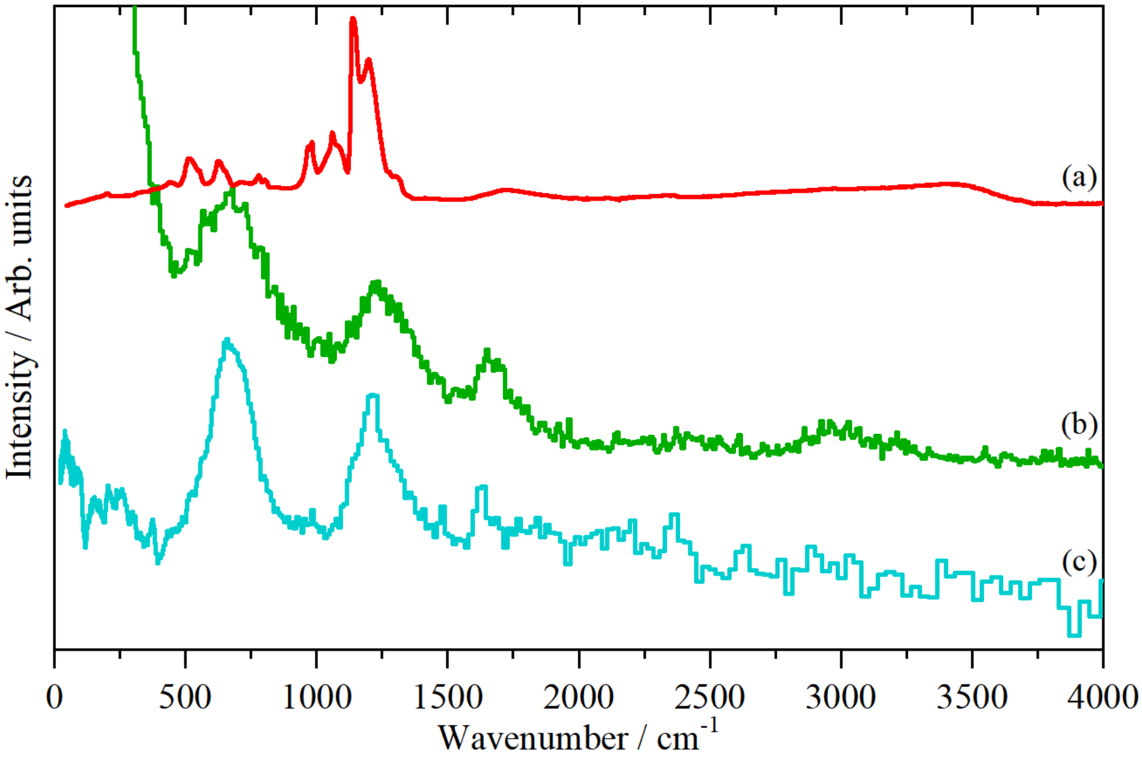

Fig. S2 Infrared (a) and INS ((b) MAPS and (c) TOSCA) of Nafion dried in a vacuum oven at 180 ˚C for two days.

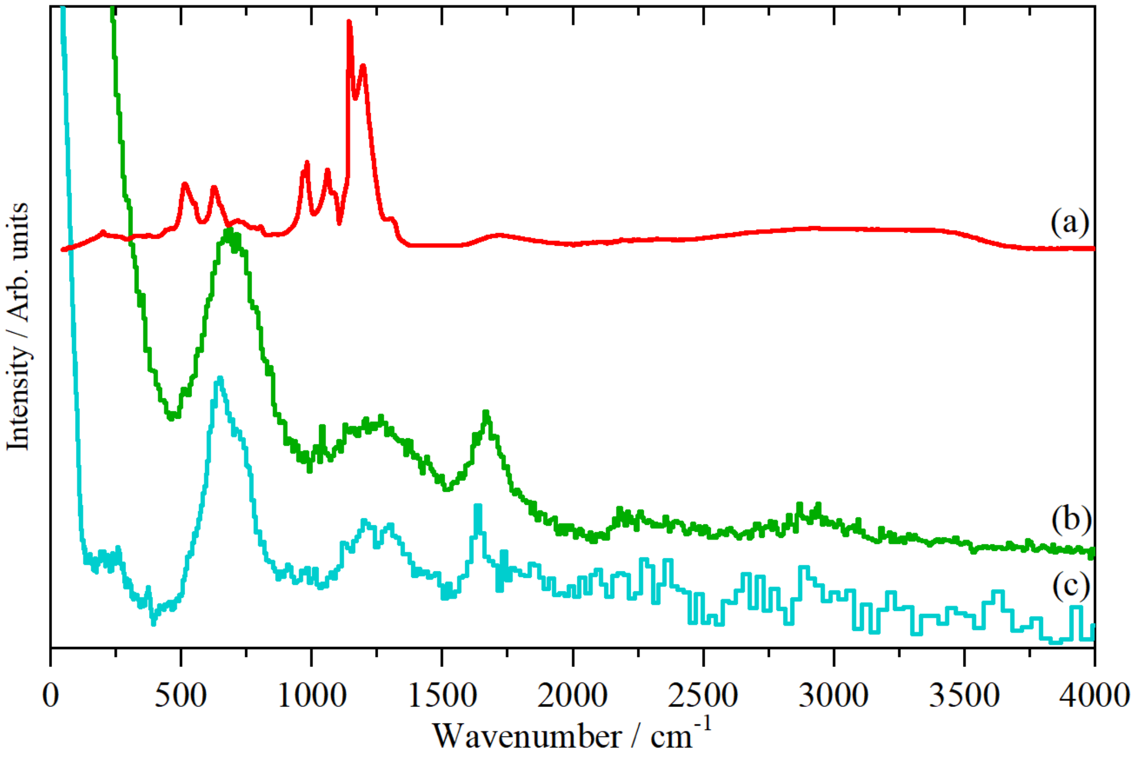

Fig. S3 Infrared (a) and INS ((b) MAPS and (c) TOSCA) of Nafion dried in a glove box at room temperature for seven days.

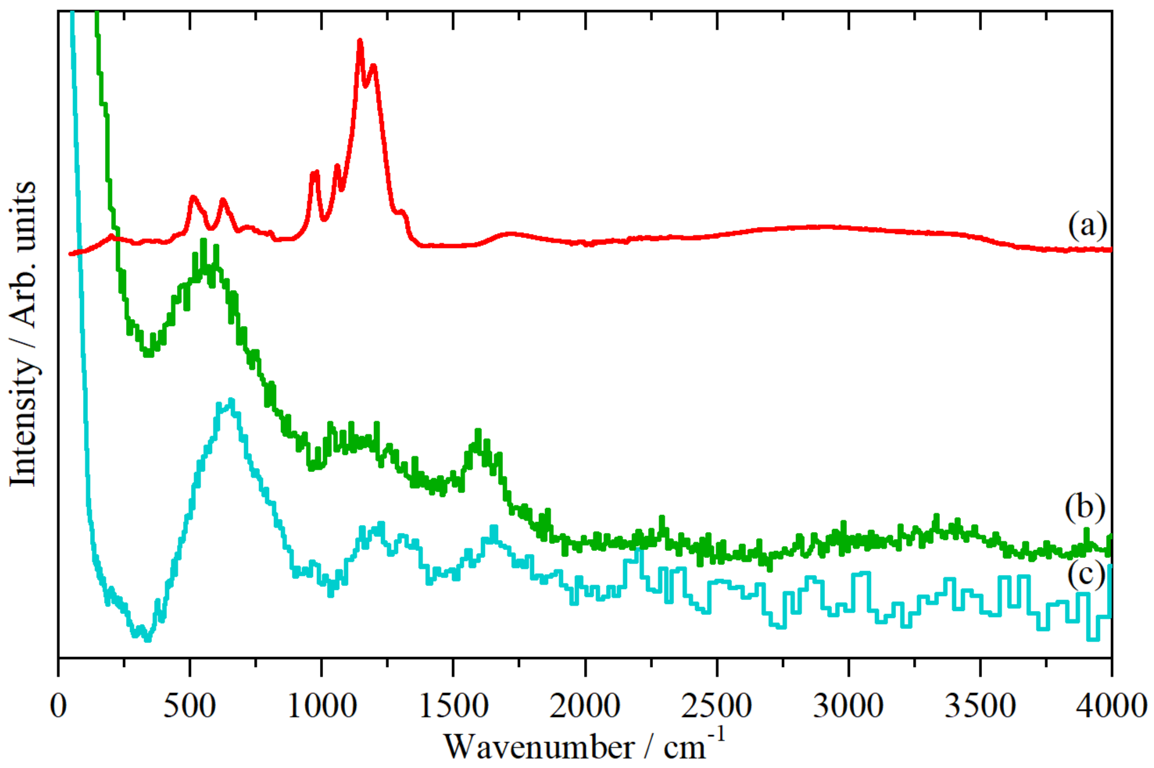

Fig. S4 Infrared (a) and INS ((b) MAPS and (c) TOSCA) of Nafion dried over P_2O_5 for 6 days.

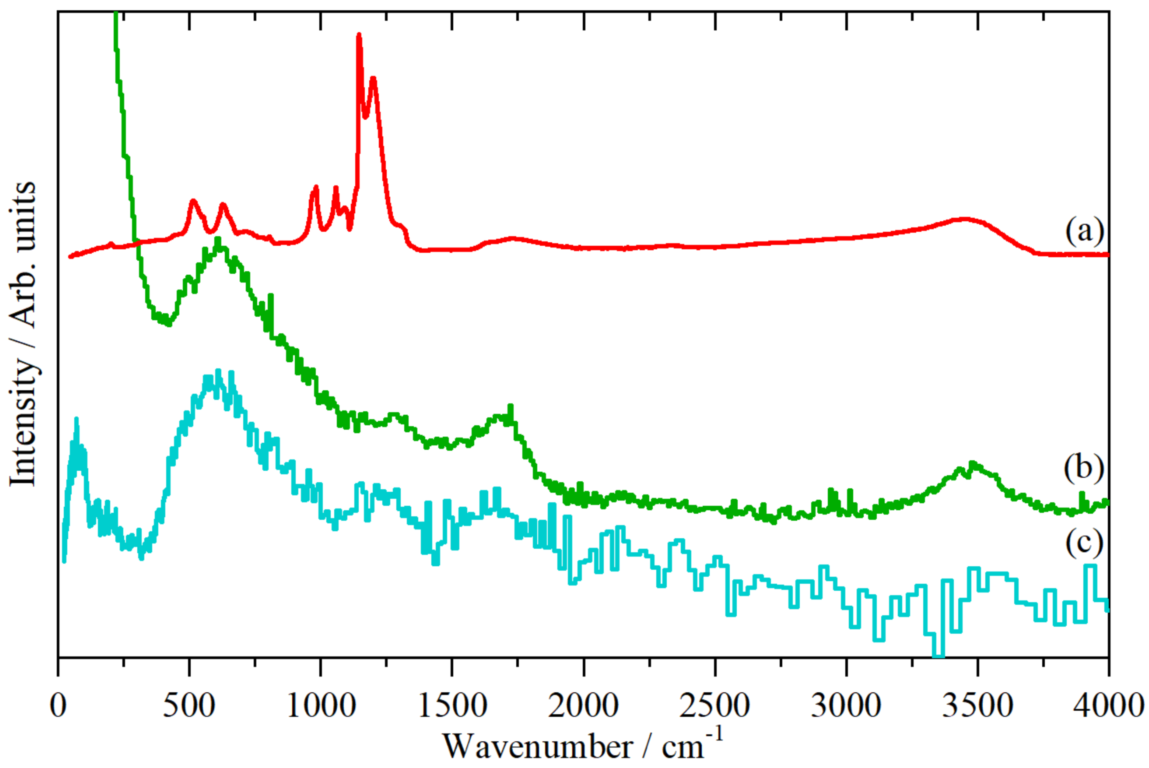

Fig. S5 Infrared (a) and INS ((b) MAPS and (c) TOSCA) of Nafion dried over $MgCl_2$ for 6 days.

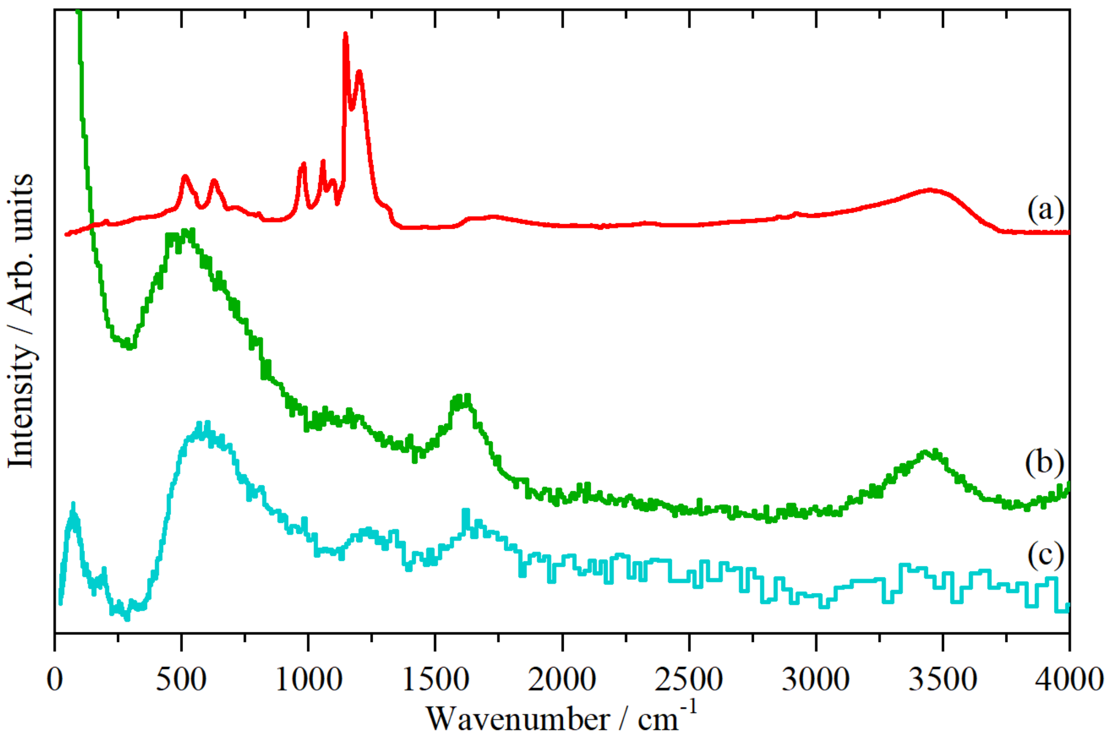

Fig. S6 Infrared (a) and INS ((b) MAPS and (c) TOSCA) of Nafion as received.

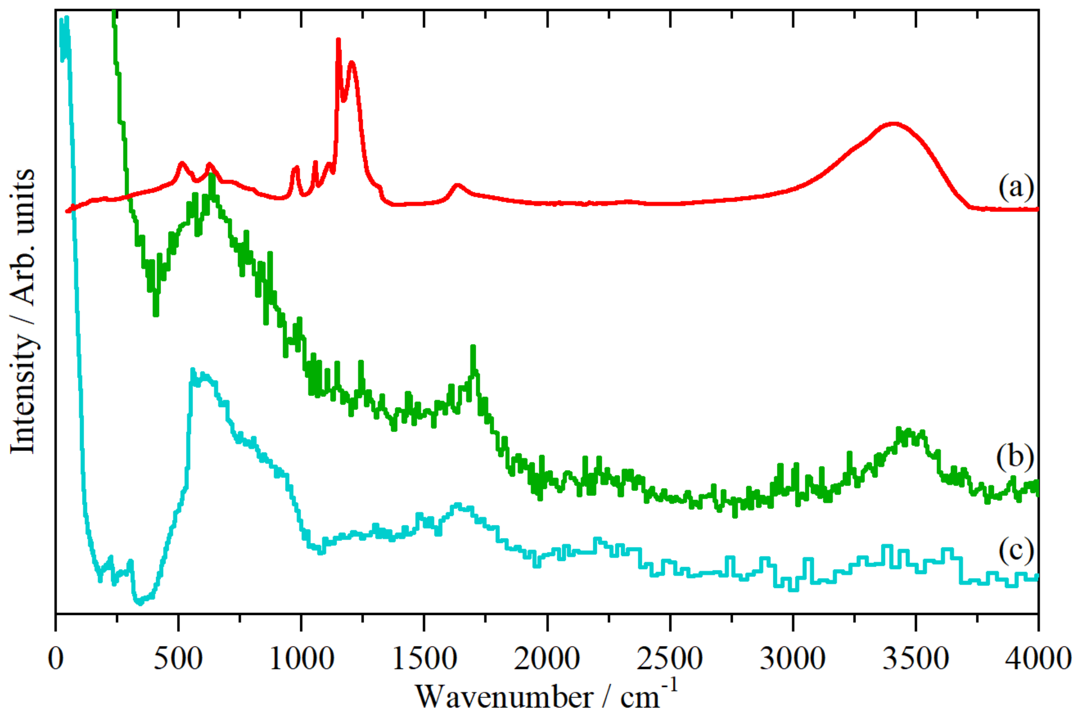

Fig. S7 Infrared (a) and INS ((b) MAPS and (c) TOSCA) of Nafion soaked in distilled water for three days.

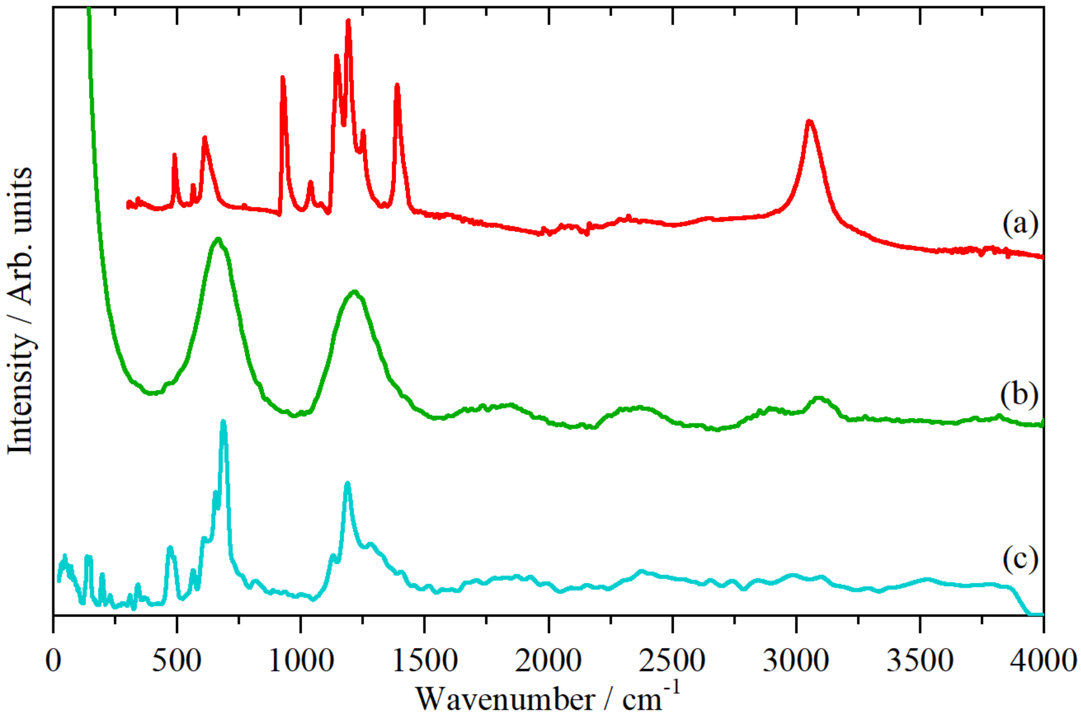

Fig. S8 Solid state infrared (a) and INS ((b) MAPS and (c) TOSCA) of TFSA.

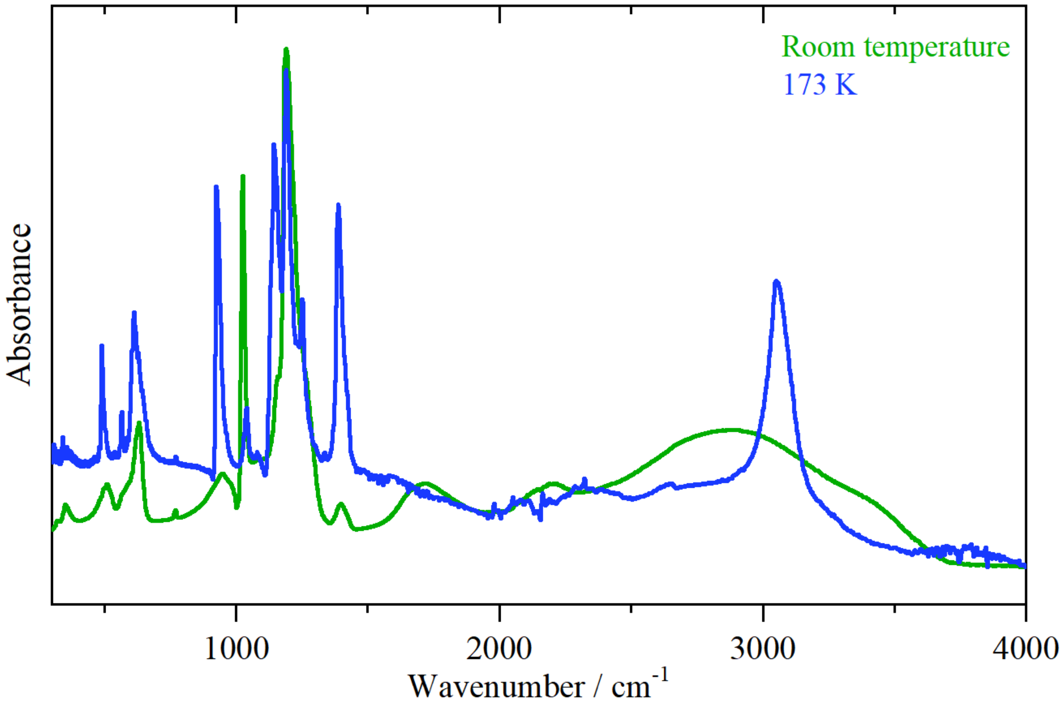

Fig. S9 Infrared spectra of liquid (room temperature, green) and solid (173 K, blue) TFSA.

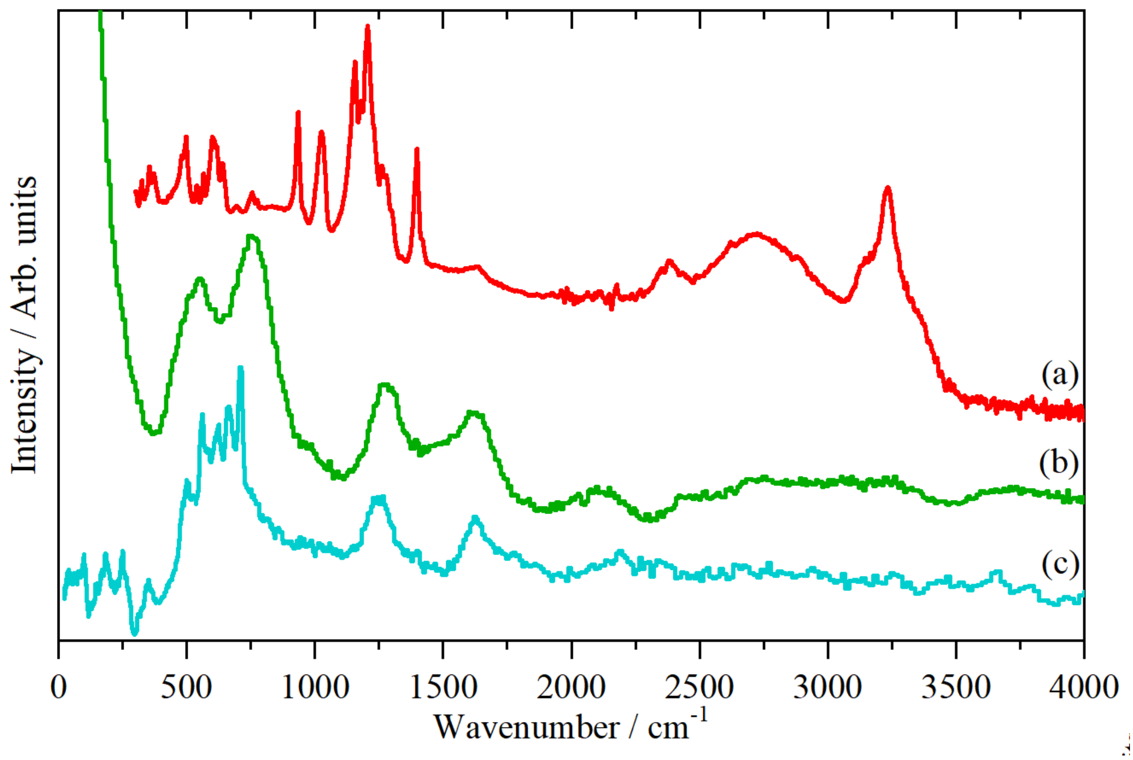

Fig. S10 Solid state infrared (a) and INS ((b) MAPS and (c) TOSCA) of TFSA.0.5H_2O.

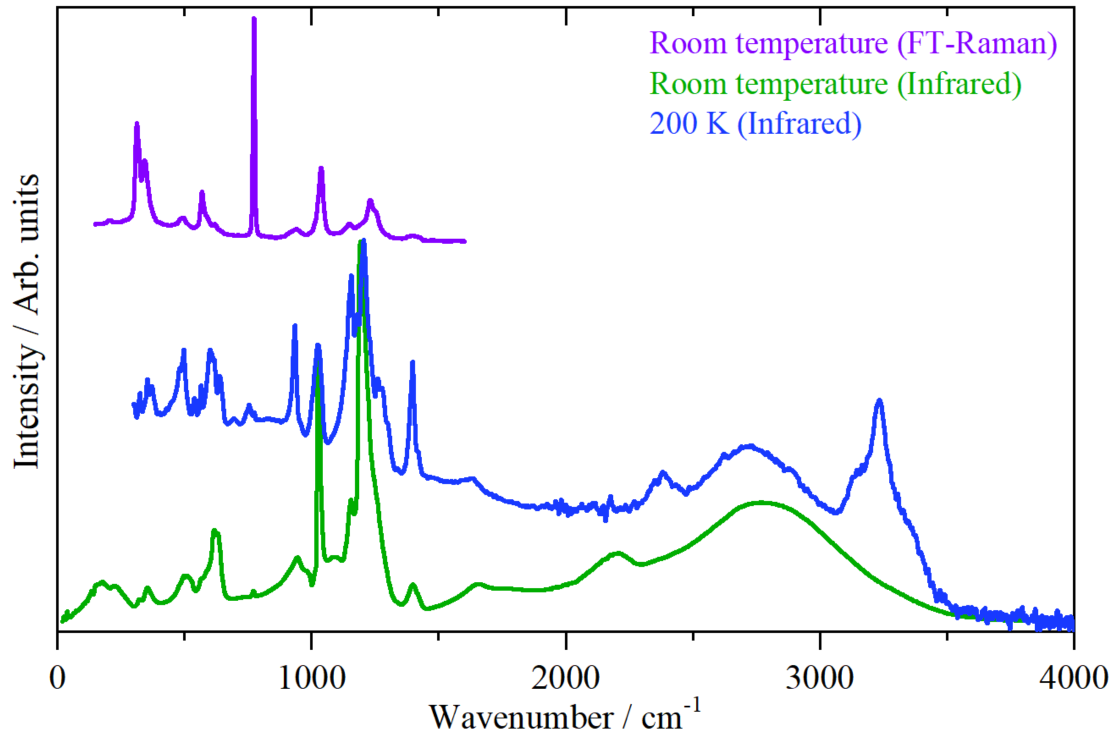

Fig. S11 Spectra of liquid (room temperature, FT-Raman violet, infrared green) and solid (200 K, infrared blue) TFSA.0.5H_2O.

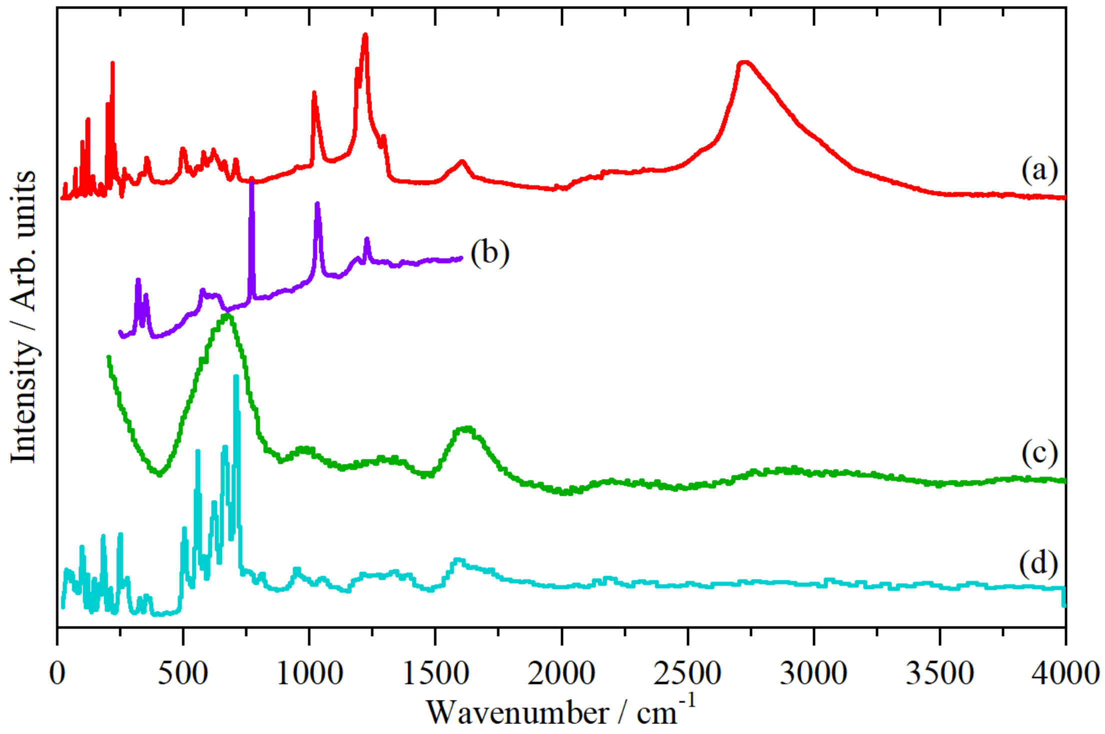

Fig. S12 Solid state infrared (a). (b) FT-Raman and INS ((c) MAPS and (d) TOSCA) spectra of TFSA.H_2O.

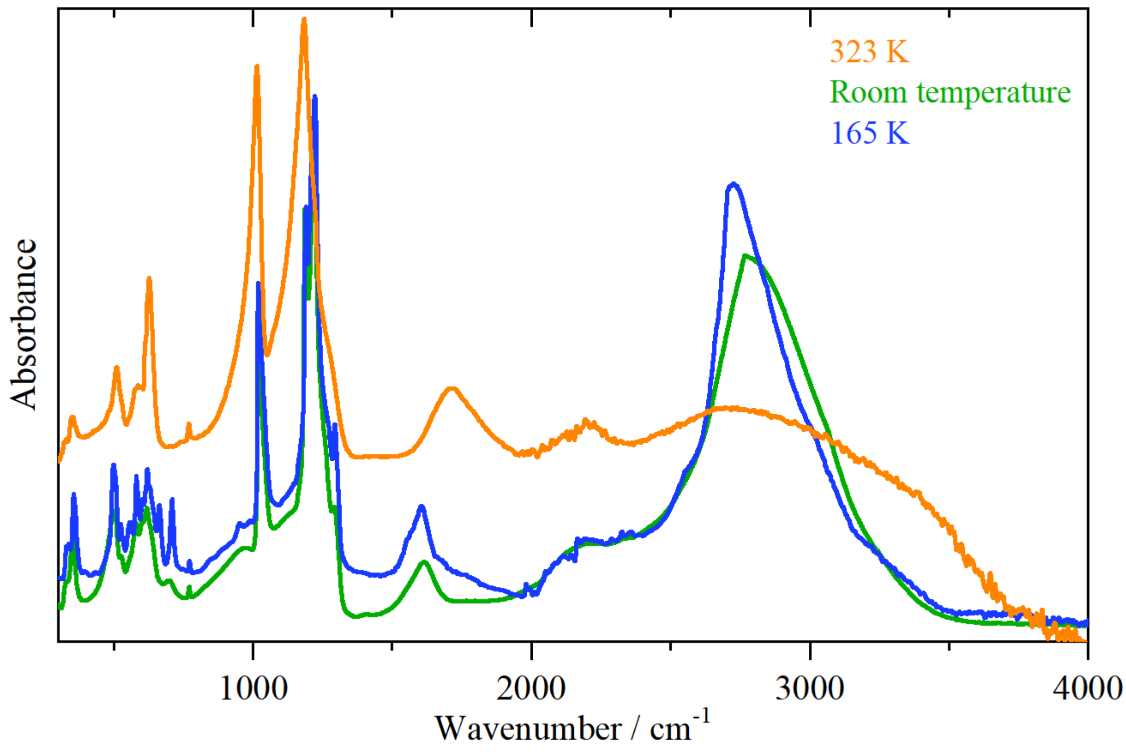

Fig. S13 Infrared spectra of liquid (323 K, orange) and solid (room temperature, green and 165 K, blue) TFSA.H_2O.

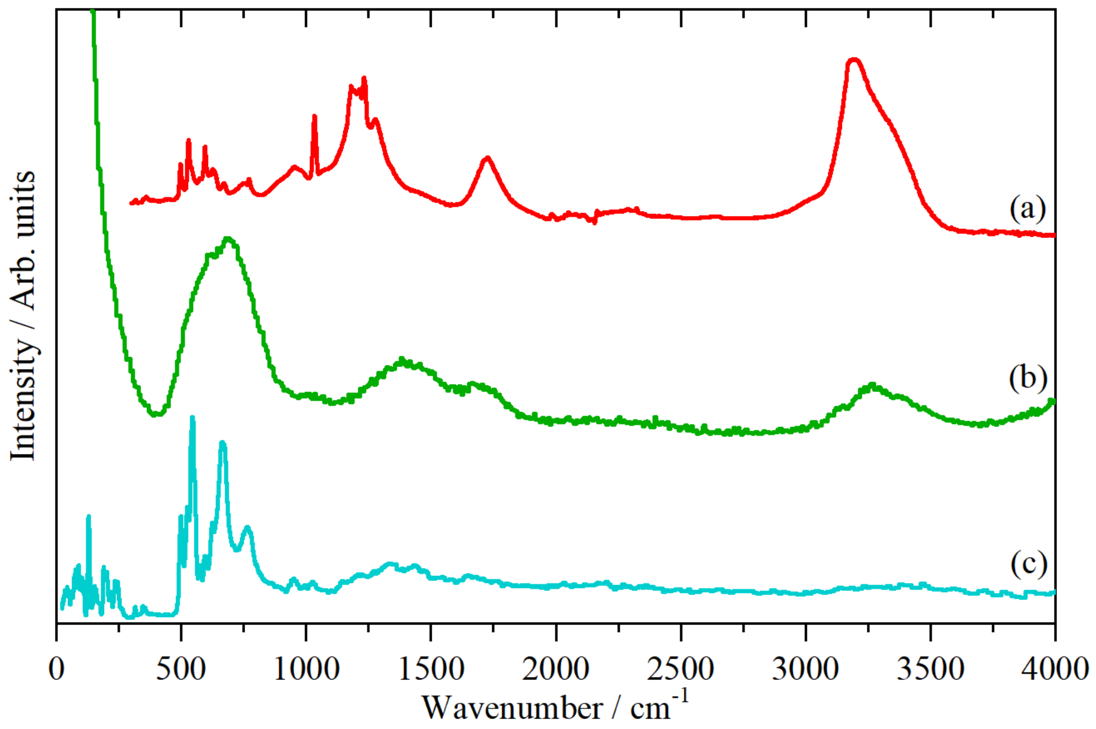

Fig. S14 Solid state infrared (a) and INS ((b) MAPS and (c) TOSCA) spectra of TFSA.2H_2O.

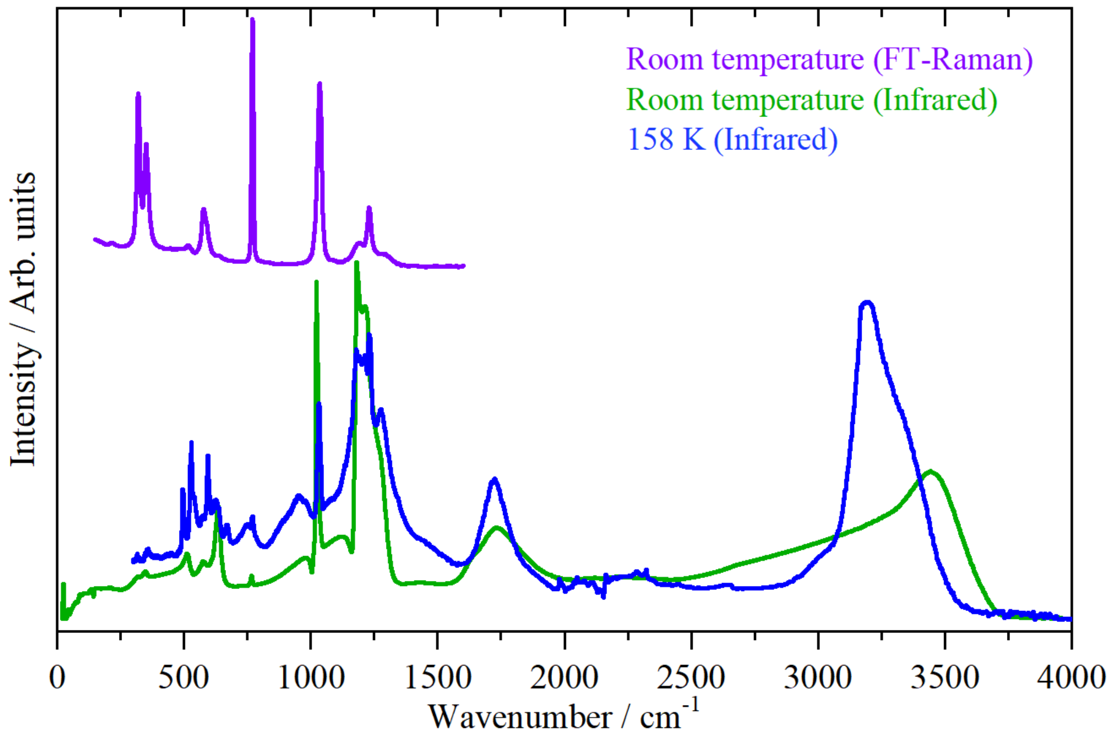

Fig. S15 Spectra of liquid (room temperature, FT-Raman violet, infrared green) and solid (158 K, infrared blue) TFSA.2H_2O.

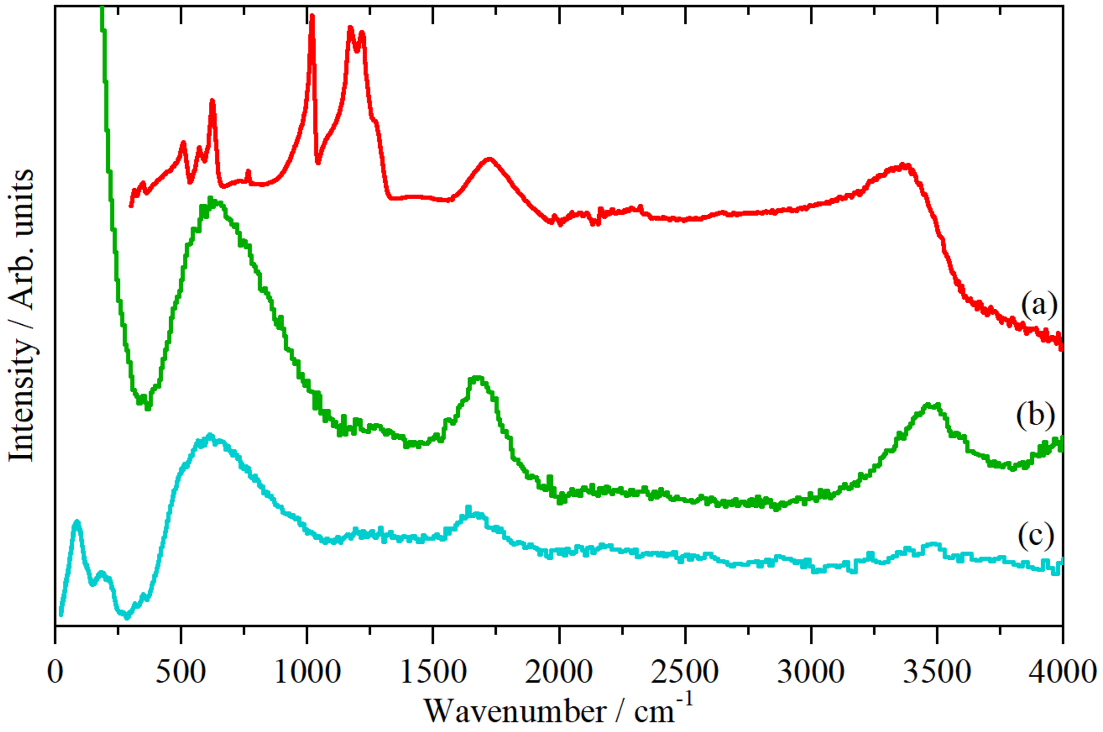

Fig. S16 Solid state infrared (a) and INS ((b) MAPS and (c) TOSCA) spectra of TFSA.4H_2O.

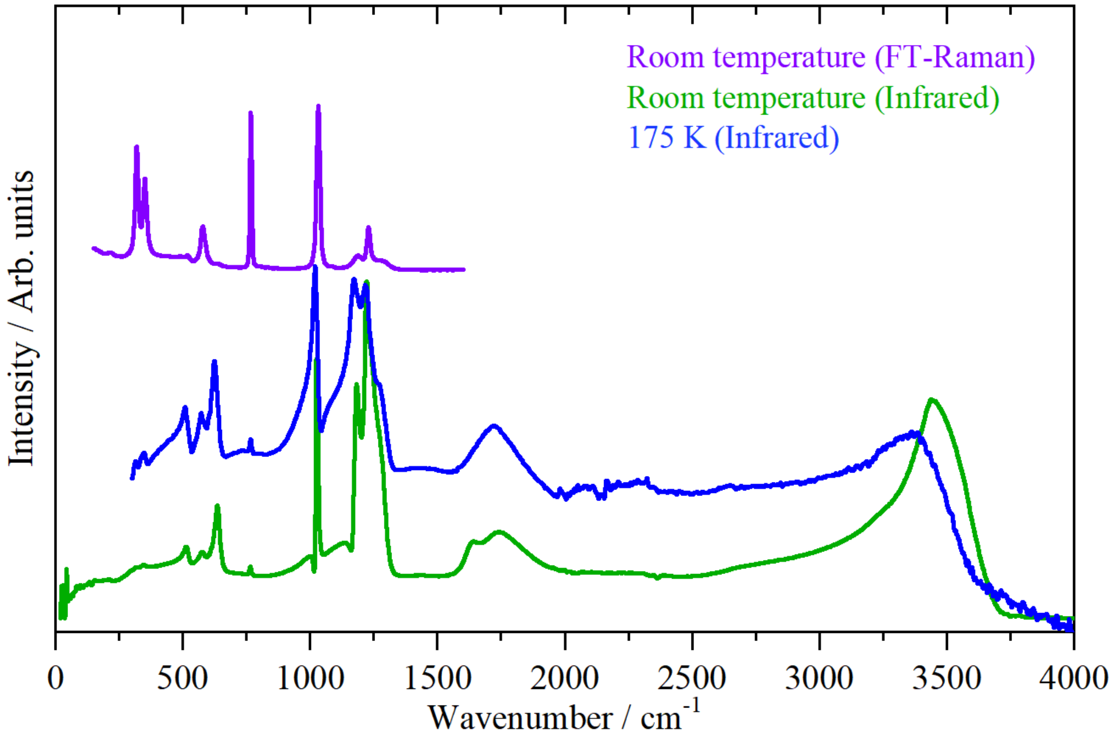

Fig. S17 Spectra of liquid (room temperature, FT-Raman violet, infrared green) and solid (158 K, infrared blue) TFSA.4H_2O.

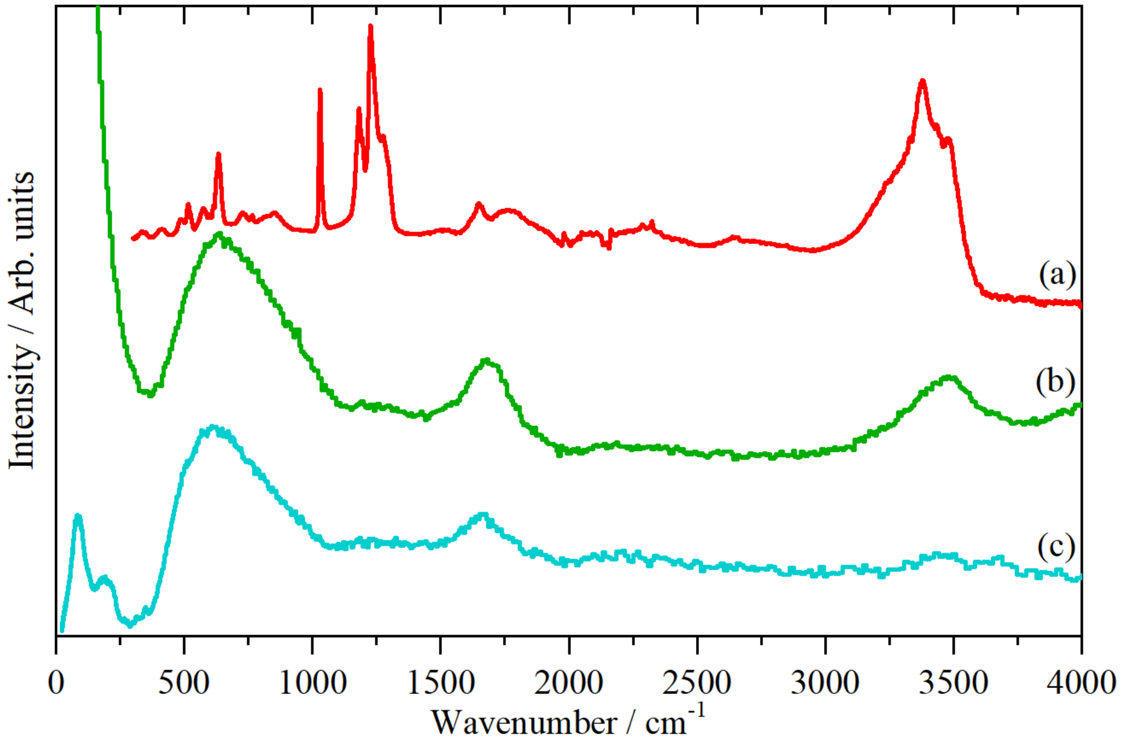

Fig. S18 Solid state infrared (a) and INS ((b) MAPS and (c) TOSCA) spectra of TFSA.5H_2O.

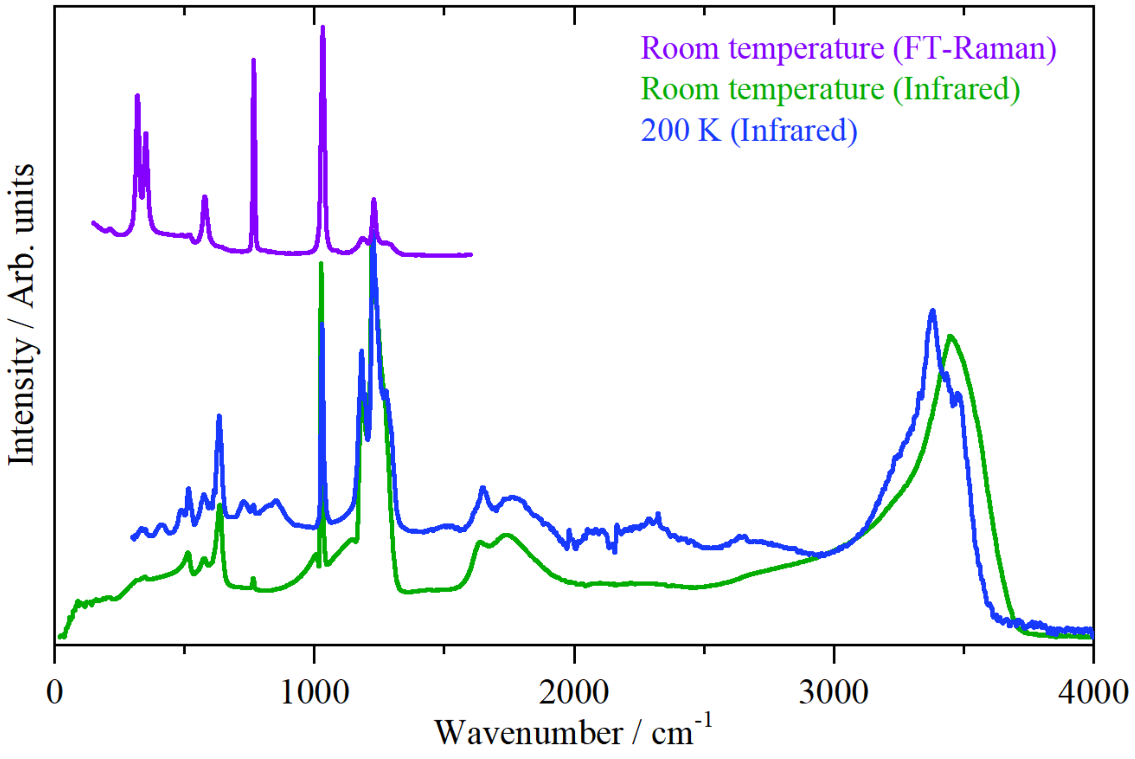

Fig. S19 Spectra of liquid (room temperature, FT-Raman violet, infrared green) and solid (200 K, infrared blue) TFSA.5H_2O.
